# Non-alcoholic fatty liver disease in polycystic ovary syndrome women

**DOI:** 10.1038/s41598-021-86697-y

**Published:** 2021-03-29

**Authors:** Young Bin Won, Seok Kyo Seo, Bo Hyon Yun, SiHyun Cho, Young Sik Choi, Byung Seok Lee

**Affiliations:** 1 Department of Obstetrics and Gynecology, Severance Hospital, Yonsei University College of Medicine, 50 Yonsei-ro, Seodaemun-gu, Seoul, 03722 Republic of Korea; 2 Institute of Women’s Life Science, Yonsei University College of Medicine, Seoul, Korea; 3 Department of Obstetrics and Gynecology, Gangnam Severance Hospital, Yonsei University College of Medicine, Seoul, Korea

**Keywords:** Diseases, Endocrinology, Medical research

## Abstract

To evaluate risk factors leading to non-alcoholic fatty liver disease (NAFLD) occurrence in polycystic ovarian syndrome (PCOS) women. A retrospective cohort study of a total of 586 women diagnosed with PCOS aged 13–35 years at the gynecology department at a university hospital was done to evaluate PCOS phenotype, metabolic syndrome (MetS) diagnosis, body composition, insulin sensitivity, sex hormones, lipid profile, liver function, and transient elastography (TE). In PCOS women with NAFLD compared to those without, MetS diagnosis (Hazard ratio [HR] 5.6, 95% Confidence interval [CI] 2.2–14.4, *p* < 0.01) and hyperandrogenism (HA) (HR 4.4, 95% CI 1.4–13.4, *p* = 0.01) were risk factors significantly associated with subsequent NAFLD occurrence, whereas 2-h insulin level in 75 g glucose tolerance test (GTT) (HR 1.2, 95% CI 0.5–2.5, *p* = 0.70) and body mass index (BMI) > 25 kg/m^2^ (HR 2.2, 95% CI 0.6–8.0, p = 0.24) was not. Among NAFLD patients who underwent TE, a higher number of MetS components indicated a worse degree of fibrosis and steatosis. MetS diagnosis and HA at PCOS diagnosis were risk factors associated with NAFLD, while 2-h insulin level in 75 g GTT and obesity were not. Although elevated aspartate aminotransferase levels were significant for NAFLD risk, liver enzyme elevations may not be present until late liver damage. Further prospective studies of PCOS women with MetS or HA are warranted to determine whether patients without liver enzyme elevations should undergo preemptive liver examinations.

## Introduction

Polycystic ovary syndrome (PCOS) is a common endocrine disorder in reproductive age women and is characterized by irregular menstruation, clinical or biochemical hyperandrogenism (HA), and polycystic ovarian morphology on ultrasonography^1–3^. It is an endocrine disorder whose association with metabolic problems is notable. There are numerous studies that demonstrate the relationship with type II diabetes mellitus (DM) and metabolic syndrome with PCOS^4,5^. In particular, the increase in insulin resistance promotes androgen production in ovarian theca cells leading to aggravation of HA^6,7^. Metabolic disturbances such as DM and obesity threaten the health of PCOS women and preventing this at an early stage is crucial from a public healthcare standpoint. Metabolic disturbances in the relatively young reproductive age women may also result in complications during pregnancy and delivery^8^. Being exposed to the hyperandrogenic environment of PCOS for a longer time could in effect be associated with a higher chance of metabolic complications later on.

Non-alcoholic fatty liver disease (NAFLD) encompasses an extensive range of liver diseases, including liver steatosis, non-alcoholic steatohepatitis, liver fibrosis, and liver cirrhosis, which may develop into liver failure and even hepatocellular carcinoma^9^. Androgens such as testosterone, dihydrotestosterone, and dehydroepiandrosterone (DHEA) are recognized as pro-apoptotic agents that act on peripheral cells such as hepatocytes^10^. The overproduction of these androgens promotes an androgen-dependent pro-apoptotic PCOS environment that may directly contribute to liver disease progression. The emphasis between the risk factors leading to NAFLD such as insulin resistance, central obesity, hypertension, and dyslipidemia and their involvement with PCOS is being highlighted in recent times^6^. Studies investigating the relationship between NAFLD and PCOS have revealed that NAFLD was more prevalent in girls with PCOS than in those without, with a prevalence of 36% up to a high of 70% PCOS patients with concurring NAFLD^11–15^.

Although the association of PCOS and NAFLD has been presented in several studies, their pathophysiology and risk factors are not clear^16–19^. It is unclear which features of PCOS increase the risk of NAFLD, particularly if the risk of NAFLD is higher in all PCOS patients. Thus, this study was conducted to evaluate risk factors associated with NAFLD occurrence to determine better identifiers in screening for metabolic abnormalities at the time of PCOS diagnosis and consider its underlying cause.

## Materials and methods

### Study population

In this retrospective cohort study, 586 women diagnosed with PCOS aged 13–35 years from January 2010 to April 2018 at the Department of Obstetrics and Gynecology in Severance Hospital, Seoul, Korea, were evaluated. Patients were included if they were diagnosed with PCOS. PCOS for adults was defined according to the 2003 Rotterdam ESHRE/ASRM-sponsored PCOS consensus workshop criteria^1^ as endorsed by the most recent international evidence-based guideline^20^. In adolescents, PCOS was defined according to the diagnosis criteria suggested by Androgen excess and PCOS society in 2009^3^, which specified oligomenorrhea and HA as a prerequisite for PCOS diagnosis^21^. Adolescent was defined as those aged between 10 to 19 years old, and adult was defined as those above 20 years old^22^. NAFLD was defined as simple fatty liver, nonalcoholic steatohepatitis, and/or liver fibrosis^23^ after excluding other—viral, alcoholic, iatrogenic by medication—causes of liver diseases.

**Figure 1:**
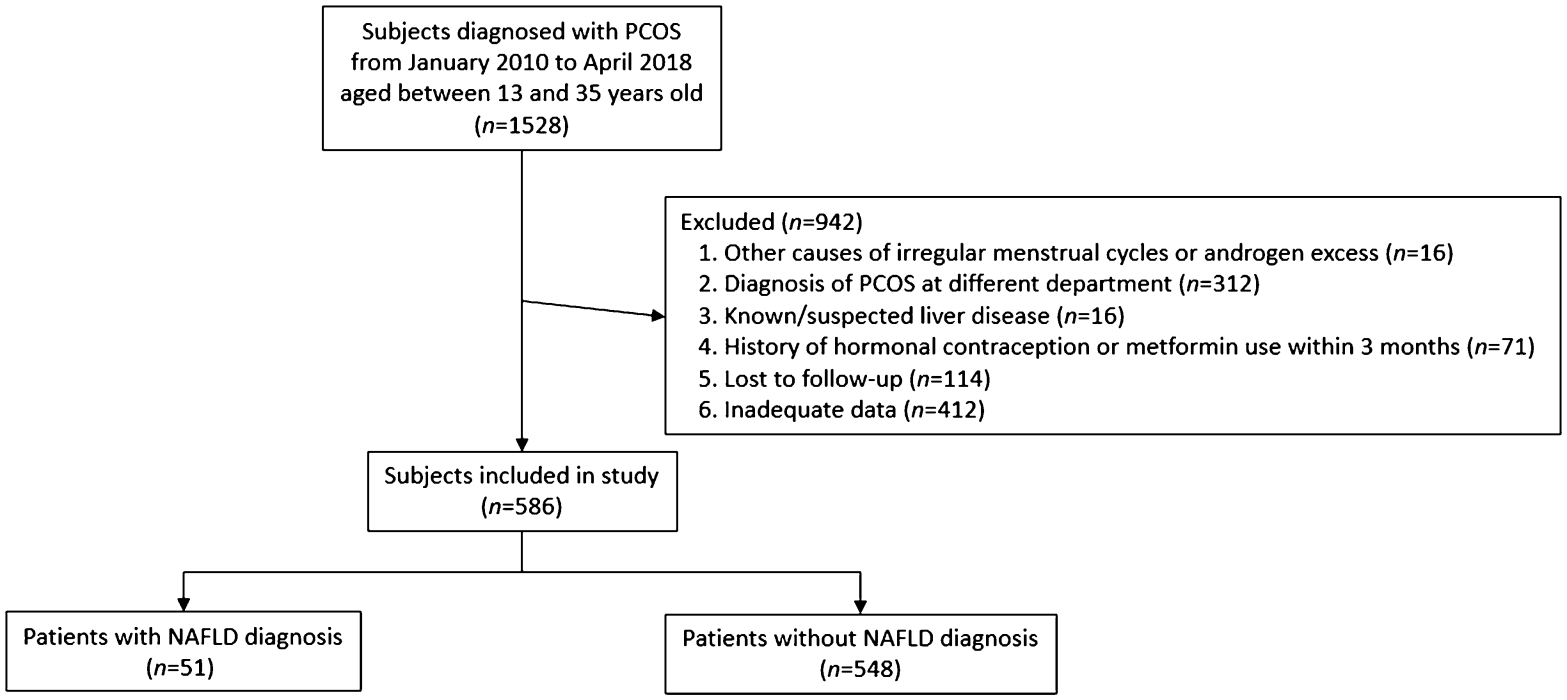
Flow chart of patient inclusion.

Patients were excluded from the analysis if they had any of following exclusion criteria: other causes of irregular menstrual cycles or androgen excess including hyperprolactinemia, uncontrolled thyroid disease, congenital adrenal hyperplasia, Cushing’s disease, androgen secreting tumor, or pregnancy; who were diagnosed with PCOS at a department apart from the Department of Obstetrics and Gynecology; anyone with health conditions that could influence liver function; a history of hormonal contraception or metformin use within 3 months preceding the diagnosis of PCOS or NAFLD; and those with inadequate data or no follow-up (Fig. 1). Unfortunately, patients diagnosed with PCOS at another department usually did not undergo gynecologic ultrasound or other studies regarding this diagnosis and bias from this exclusion was unavoidable.

### Measures

Demographic and physical characteristics such as age, body mass index (BMI), hypertension diagnosis, NAFLD diagnosis, MetS diagnosis, and PCOS phenotype were investigated. Laboratory examination results including aspartate aminotransferase (AST, reference: normal ≤ 43 U/L), alanine aminotransferase (ALT, reference: normal ≤ 45 U/L), total cholesterol, triglyceride, high-density lipoprotein (HDL), low-density lipoprotein (LDL), dehydroepiandrosterone sulfate (DHEA-S), total testosterone, sex hormone binding globulin (SHBG), and anti-Mullerian hormone (AMH) levels; 75 g oral glucose tolerance test (fasting and 2-h glucose and insulin); homeostasis model assessment of insulin resistance (HOMA-IR); fasting glucose insulin ratio (FGIR); and free androgen index (FAI) were examined. The standard values were determined by the laboratory references of our institution.

The 75 g oral glucose tolerance testing (GTT) was routinely performed in all patients diagnosed with PCOS to evaluate insulin resistance. HOMA-IR was calculated as [fasting glucose (mg/dL) × basal insulin (µUI/mL)]/405. Patients were considered to have MetS if ≥ 3 of the following criteria, based on the criteria presented by the National Cholesterol Education Program/Adult Treatment Panel III (NCEP/ATP III) and adapted for patients with PCOS by the 2003 Rotterdam ESHRE/ASRM-sponsored PCOS consensus workshop, were satisfied: (1) abdominal obesity as waist circumference of > 88 cm, (2) elevated triglyceride level of ≥ 150 mg/dL, (3) low HDL cholesterol level of < 50 mg/dL, (4) elevated blood pressure of ≥ 130/ ≥ 85 mmHg, and (5) impaired fasting glucose level of 110–126 mg/dL and/or 2-h postprandial glucose level of 140–199 mg/dL^1^. Regrettably, because waist circumference was not routinely checked in our study, BMI was used as an alternative^24^. The cut-off for obesity in an Asian population was defined as BMI of ≥ 25 kg/m^2^^25^. Those with BMI ≤ 20 kg/m^2^ was defined as lean, 20 < BMI < 25 kg/m^2^ as normal, BMI ≥ 25 kg/m^2^ as obese. Diagnosis of NAFLD was made by hepatologist: after referral if indicated by an abnormal AST or ALT level or incidentally found during other evaluation-for example, computed tomography or abdominal sonography performed at emergency room or health check-up. NAFLD was diagnosed using at least 2 non-invasive screening methods such as abdominal sonography, transient elastography (TE; FibroScan, Echosens, Paris, France), and magnetic resonance imaging^26^. Controlled attenuation parameter was used as a parameter for liver steatosis in those undergone TE. TE showed liver stiffness measurements for fibrosis in stage 0 < 5.5 kPa, stage 1 5.5–7.4 kPa, stage 2 7.5–9.4 kPa, stage 3 9.5–10.9 kPa, and stage 4 > 11 kPa. The parameters for liver steatosis was stage 0 < 238 dB/m, stage 1 238–259 dB/m, stage 2 260–292 dB/m, and stage 3 > 293 dB/m.

### Statistical analysis

All statistical analyses were performed using SPSS ver. 23 (SPSS Inc., Chicago, IL, USA). Baseline patient characteristic data were analyzed using Student’s t-test or chi-squared test. Continuous variables are presented as mean (standard deviation), and categorical variables are presented as numbers (percentage). Cox regression analysis was performed to determine risk factors according to the diagnosis of NAFLD. Univariate analysis was performed to assess the relationship between each variable and NAFLD. Data were corrected through multivariate analysis. A *p* value of < 0.05 was considered statistically significant.

### Ethics approval

This study was approved by the Yonsei University Health System, Severance Hospital’s Institutional Review Board (4-2018-0786). All procedures performed in the study were done in accordance with the ethical standards of the institutional review board and with the 1964 Helsinki declaration and its later amendments, or comparable ethical standards. Informed consent was exempted by the Institutional Review Board for it’s nature of retrospective study.

## Results

### NAFLD occurrence and PCOS patient characteristics

Of the 1528 women diagnosed with PCOS during the study duration, 586 were finally included. 12 adolescents (11.8%) and 39 adults (8.1%) were diagnosed with NAFLD (total 51 patients, 8.7%) during their follow up. All data results were obtained when the diagnosis of PCOS was made, exception of liver sonography and TE results, which were obtained during NAFLD diagnosis. TE was performed in only 28 patients, all of whom presented with elevated AST/ALT levels. The remaining 23 patients diagnosed with NAFLD underwent abdominal ultrasonography, which is the gold standard for non-invasive diagnosis of NAFLD. 1 patient who underwent abdominal ultrasonography additionally received MRI for confirmation. The average follow-up period for the total PCOS population was 36.8 months. The median follow-up length of NAFLD patients was 37 (range 0–143) months and that for non-NAFLD patients was 26 (range 0–118) months. 17 patients (total 51 patients with NAFLD, 33.3%) were diagnosed with NAFLD before the diagnosis of PCOS was made. Excluding these patients, an average of 15.1 months elapsed from the point of PCOS diagnosis to the occurrence of NAFLD.

### Characteristics of NAFLD occurrence in PCOS patients

Baseline characteristics comparing PCOS women with and without NAFLD are shown in Table 1. MetS diagnosis rate was significantly higher in women with NAFLD than in those without NAFLD. AST and ALT level elevation was also significantly correlated with NAFLD. Indices reflecting insulin resistance including 2-h 75 g GTT insulin, FGIR, HOMA-IR were markedly higher in NAFLD group. Of the 51 NAFLD patients, 43 (84.3%) had HA as opposed to 345 of 535 patients (64.5%) without NAFLD, which was significant (*p* = 0.01). Total testosterone and DHEA-S levels were not statistically significant. Mean BMI of our study population was 23.83 kg/m^2^, showing higher proportion of non-obese PCOS patients (n = 387, 66%) compared to obese PCOS patients (n = 199, 34%) with significant difference (p < 0.001). Proportion of obese PCOS was significantly higher in NAFLD compared to the non-NAFLD group (p < 0.001).

**Table 1:** Baseline characteristics of PCOS women according to subsequent NAFLD occurrence.

Variables	NAFLD (n = 51)	Non-NAFLD (n = 535)	*p* value
Age at PCOS diagnosis (year)	25 (13–35)	24 (12–35)	0.94
Adolescent PCOS (n)	12 (23.5)	90 (16.8)	0.22
**BMI (kg/m**^**2**^**)**	29.3 (± 5.19)	23.3 (± 4.95)	< 0.01
Lean (n)	2 (3.9)	150 (28.0)	< 0.01
Normal (n)	8 (15.7)	228 (42.6)	
Obese (n)	41 (80.4)	157 (29.4)	
AST (IU/L)	28 (10–137)	16 (7–119)	< 0.01
ALT (IU/L)	45 (6–259)	13 (1–298)	< 0.01
Total cholesterol (mg/dL)	204 (146–301)	178 (111–348)	< 0.01
Triglycerides (mg/dL)	177 (72–586)	78 (28–2130)	< 0.01
HDL (mg/dL)	42 (25–66)	58 (26–106)	< 0.01
LDL (mg/dL)	123.4 (16.6–202.8)	100.0 (11.2–261.2)	< 0.01
Fasting glucose (mg/dL)	97 (77–226)	90 (66–229)	< 0.01
2-h 75 g GTT glucose (mg/dL)	132 (77–356)	102 (50–414)	< 0.01
Fasting insulin (μU/mL)	22.7 (± 11.8)	11.6 (± 11.7)	< 0.01
2-h 75 g GTT insulin (μU/mL)	109.6 (8.9–537.7)	48.08 (1.8–1000.0)	< 0.01
FGIR	5.6 (1.1–35.2)	10.5 (0.6–78.6)	< 0.01
HOMA-IR	5.3 (0.5–17.9)	2.0 (0.1–22.3)	< 0.01
Total testosterone (ng/dL)	45.9 (± 23.0)	44.2 (± 22.7)	0.63
SHBG (nmol/L)	19.1 (6.4–127.3)	47.4 (6.8–200.0)	< 0.01
FAI (%)	6.7 (1.2–45.5)	3.1 (0–37.2)	< 0.01
DHEA-S (μg/dL)	237.2 (± 113.8)	233.4 (± 100.5)	0.82
LH (mIU/mL)	7.3 (1.4–31.1)	9.0 (0.4–43.6)	< 0.01
FSH (mIU/mL)	5.8 (± 1.5)	6.2 (± 2.0)	0.10
Estradiol (pg/mL)	56.7 (± 65.9)	54.7 (± 50)	0.79
AMH (ng/mL)	6.9 (2.1–19.6)	11.3 (0.3–38.3)	< 0.01
HA (n)	43 (84.3)	345 (64.5)	0.01
MetS diagnosis (n)	32 (62.7)	56 (10.5)	< 0.01
**Number of MetS components (n)**			< 0.01
0	2 (3.9)	274 (51.2)	
1	7 (13.7)	135 (25.2)	
2	10 (19.6)	70 (13.1)	
≥ 3	32 (62.7)	56 (10.5)	
Liver steatosis (dB/m)	328.9 (185.0–390.0)	NA	
Liver fibrosis (kPa)	7.7 (3.1–17.8)	NA	

Values are shown as median (minimum–maximum), mean (± standard deviation), or number (%).

*ALT* alanine aminotransferase, *AMH* anti-Müllerian hormone, *AST* aspartate transaminase, *BMI* body mass index, *DHEA-S* dehydroepiandrosterone sulfate, *FGIR* fasting glucose insulin ratio, *FSH* follicle stimulating hormone, *GTT* glucose tolerance test, *HA* hyperandrogenism, *HDL* high-density lipoprotein, *HOMA-IR* homeostasis model assessment of insulin resistance, *LDL* low-density lipoprotein, *LH* luteinizing hormone, *MetS* metabolic syndrome, *PCOS* polycystic ovary syndrome, *SHBG* sex hormone binding globulin.

### Baseline characteristics according to the age of PCOS diagnosis

Comparing adolescent and adult PCOS patients (Table 2), BMI was significantly higher in adolescents than in adults. Lipid profile was in the normal range for both groups. Indices reflecting insulin resistance seemed to be higher in the adolescent group. 2-h 75 g GTT insulin was more elevated in adolescents. HOMA-IR was 4.3 in adolescents and 2.7 in adults (*p* < 0.01). Establishing insulin resistance as HOMA-IR > 3.16 for adolescents^27^ or > 2.5 for adults^28,29^ and 2-h 75 g GTT insulin > 41 μU/mL^30^, both parameters showed a greater insulin resistance in adolescents compared to adults. FGIR showed conflicting results with 14.0 in adults which was higher than the 7.1 for adolescents (*p* < 0.01), with FGIR < 7 as abnormal for adolescents^31^ and < 4.5 as abnormal for adults^32^. Both were not indicative of insulin resistance, although it was borderline for adolescents. The duration of time that lapsed after PCOS diagnosis to NAFLD diagnosis in adolescents compared to adults was not significant (35.6 months vs. 38.0 months, p = 0.48).

**Table 2:** Baseline characteristics according to the age of PCOS diagnosis.

Variables	Adolescent (n = 102)	Adult (n = 484)	*p* value
Age at PCOS diagnosis (year)	17 (12–19)	26 (20–35)	< 0.01
Duration between diagnosis of PCOS to NAFLD (months)	35.6 (± 30.6)	38.0 (± 31.7)	0.48
BMI (kg/m^2^)	25.5 (± 5.2)	23.5 (± 5.2)	< 0.01
AST (IU/L)	17 (9–100)	17 (9–137)	0.16
ALT (IU/L)	15 (7–259)	14 (1–298)	0.07
Total cholesterol (mg/dL)	182.2 (± 33.8)	184.5 (± 34.4)	0.55
Triglycerides (mg/dL)	129.1 (± 85.7)	103.7 (± 117.2)	0.04
HDL (mg/dL)	53.6 (± 13.2)	58.5 (± 14.9)	< 0.01
LDL (mg/dL)	106.1 (± 30.0)	105.2 (± 31.1)	0.78
Fasting glucose (mg/dL)	92.6 (± 15.8)	93.0 (± 15.0)	0.81
2-h 75 g GTT glucose (mg/dL)	119.9 (± 42.3)	111.1 (± 39.4)	0.04
Fasting insulin (μU/mL)	14.4 (4.3–69.1)	8.2 (0.8–118.7)	< 0.01
2-h 75 g GTT insulin (μU/mL)	91.0 (14.7–1000)	45.4 (1.8–369.4)	< 0.01
FGIR	7.1 (± 4.0)	14.0 (± 10.4)	< 0.01
HOMA-IR	4.3 (± 3.2)	2.7 (± 3.0)	< 0.01
Total testosterone (ng/dL)	53.0 (± 27.4)	42.6 (± 21.2)	< 0.01
SHBG (nmol/L)	39.3 (± 40.8)	56.9 (± 37.7)	< 0.01
FAI (%)	7.1 (0–45.5)	2.9 (0–28.1)	< 0.01
DHEA-S (μg/dL)	271.9 (± 116.3)	224.3 (± 95.4)	< 0.01
LH (mIU/mL)	11.4 (± 6.5)	10.3 (± 6.8)	0.14
FSH (mIU/mL)	5.7 (1.5–9.9)	6.3 (1.3–24.6)	0.01
Estradiol (pg/mL)	46.6 (± 30.2)	56.6 (± 54.9)	0.08
AMH (ng/mL)	12.6 (± 5.9)	11.7 (± 6.0)	0.15
HA (n)	102 (100.0)	286 (59.1)	< 0.01
MetS diagnosis (n)	17 (16.7)	71 (14.7)	0.61
**Number of MetS components**			< 0.01
0	34 (33.3)	242 (50.0)	
1	28 (27.5)	114 (23.6)	
2	23 (22.5)	57 (11.8)	
≥ 3	17 (16.7)	71 (14.7)	
Development of NAFLD (n)	12 (11.8)	39 (8.1)	0.23
Liver steatosis (dB/m)	315.4 (± 30.6)	320 (± 48.8)	0.22
Liver fibrosis (kPa)	6.6 (± 2.1)	8.4 (± 3.6)	0.82

Values are shown as median (minimum–maximum), mean (± standard deviation), or number (%).

*ALT* alanine aminotransferase, *AMH* anti-Müllerian hormone, *AST* aspartate transaminase, *BMI* body mass index, *DHEA-S* dehydroepiandrosterone sulfate, *FGIR* fasting glucose insulin ratio, *FSH* follicle stimulating hormone, *GTT* glucose tolerance test, *HA* hyperandrogenism, *HDL* high-density lipoprotein, *HOMA-IR* homeostasis model assessment of insulin resistance, *LDL* low-density lipoprotein, *LH* luteinizing hormone, *MetS* metabolic syndrome, *PCOS* polycystic ovary syndrome, *SHBG* sex hormone binding globulin.

### Relationship between MetS diagnosis and liver steatosis

**Figure 2:**
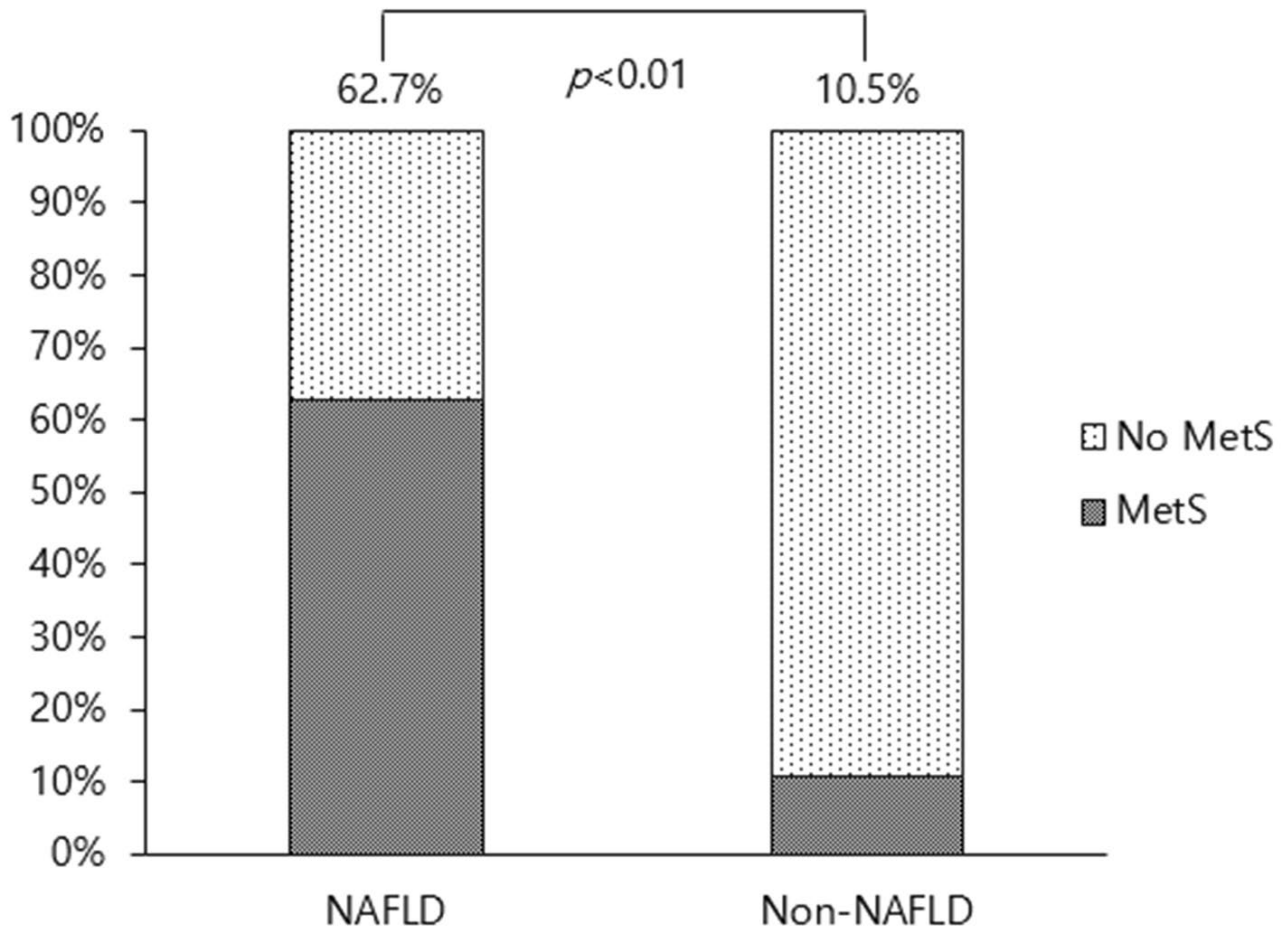
Metabolic syndrome at the time of polycystic ovary syndrome (PCOS) diagnosis in non-alcoholic fatty liver disease (NAFLD) and non-NAFLD Korean women.

**Figure 3:**
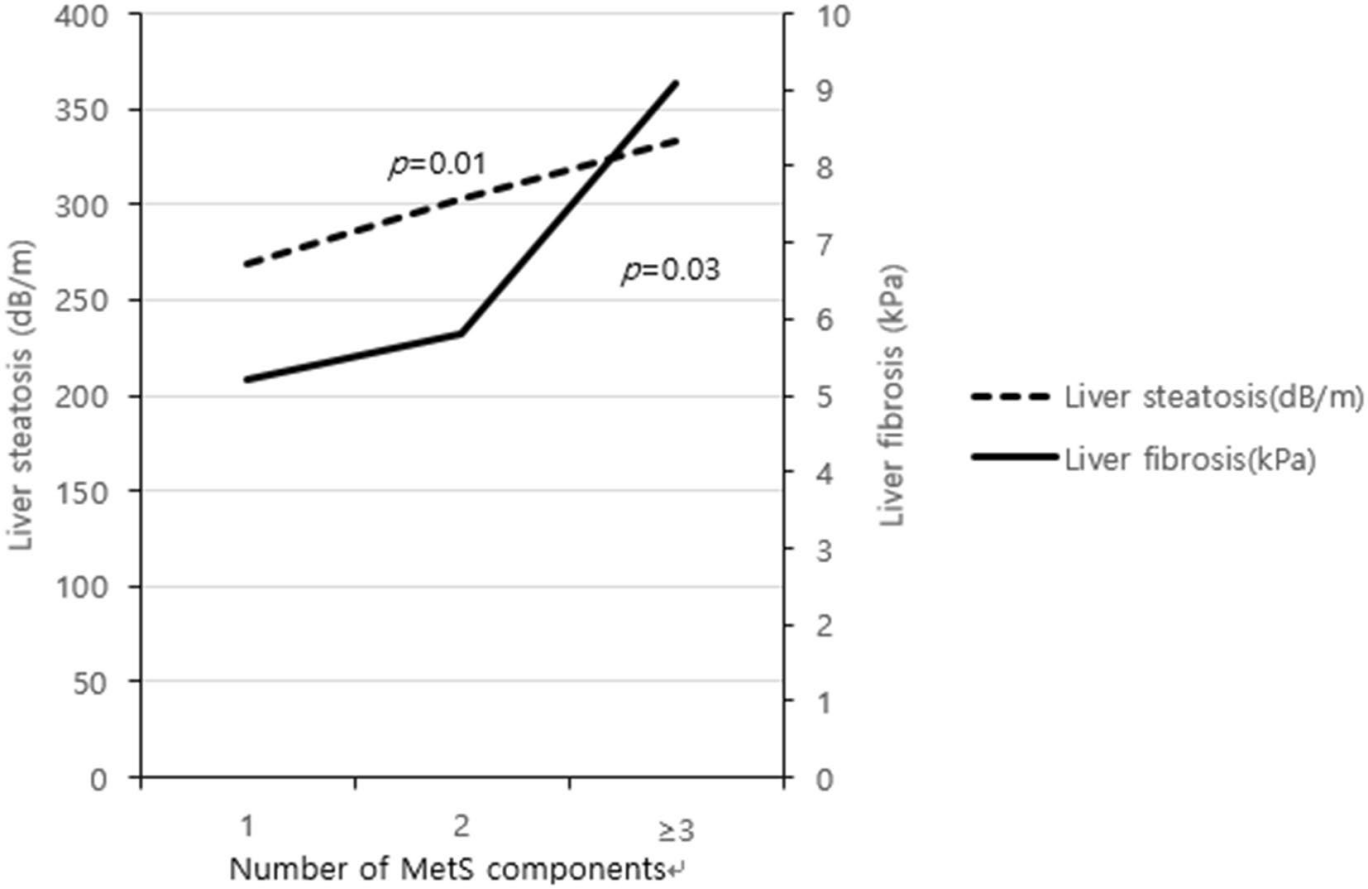
Number of metabolic syndrome components at the time of polycystic ovary syndrome diagnosis and liver fibroscan profiles in Korean women.

MetS diagnosis at the time of PCOS diagnosis was 62.7% in NAFLD patients and 10.5% in non-NAFLD patients (*p* < 0.01, Fig. 2). The proportion of NAFLD patients increased as the number of MetS components increased, which was not true for non-NAFLD patients (Table 1). Among NAFLD patients who underwent TE, the greater the number of MetS components, the worse was the degree of fibrosis and steatosis (Fig. 3). The mean stiffness scores on liver fibroscan were 328.9 dB/m, which suggests severe liver steatosis, and 7.7 kPa, which suggests significant liver fibrosis (Table 1).

Univariate cox analysis was performed for risk factors associated with NAFLD occurrence in our PCOS population (Table 3). It showed that all MetS components; hypertension, obesity, hypertriglyceridemia, low HDL level, and impaired fasting glucose level, were statistically significant. As the number of MetS components increased from 1 to ≥ 3, the hazard ratio (HR) also increased from 4.2 to 53.8. Elevated AST and ALT levels were both linked to NAFLD. Insulin resistance, portrayed as 2-h 75 g GTT insulin ≥ 100 μU/mL, FGIR < 4.5, and HOMA-IR > 2.5 were all statistically significant. HA was significant, while age at PCOS diagnosis was not. Obesity, specified as BMI > 25, compared to the normal was significant compared to lean group which was not significant.

**Table 3:** Univariate analysis of variables associated with NAFLD development in PCOS patients.

Variables	Unadjusted		
	HR	95% CI	*p* value
**MetS component**			
HTN	7.2	3.4–14.9	< 0.01
Obesity	9.6	4.2–21.9	< 0.01
Hypertriglyceridemia	17.5	8.4–36.2	< 0.01
Low HDL	5.0	2.5–10.0	< 0.01
Impaired fasting glucose	5.6	2.9–10.9	< 0.01
**Number of MetS components**			
1	4.2	0.8–22.9	0.1
2	13.3	2.8–64.1	< 0.01
≥ 3	53.8	12.6–228.8	< 0.01
**BMI 20–25 kg/m**^**2**^** (Reference)**			
BMI < 20 kg/m^2^ (Lean)	0.6	0.1–3.1	0.54
BMI > 25 kg/m^2^ (Obese)	8.1	3.2–21.0	< 0.01
Age at PCOS diagnosis	1.6	0.8–3.4	0.22
Elevated AST	21.9	11.0–43.8	< 0.01
Elevated ALT	13.9	7.1–27.5	< 0.01
2-h 75 g GTT insulin ≥ 100 µU/mL	5.2	2.7–10.3	< 0.01
FGIR < 4.5	0.8	0.8–0.9	< 0.01
HOMA-IR > 2.5	1.2	1.1–1.3	< 0.01
SHBG	0.94	0.92–0.97	< 0.01
FAI > 4.5	4.9	2.3–10.5	< 0.01
LH	1.0	0.9–1.0	0.13
AMH	0.8	0.8–0.9	< 0.01
HA	3.7	1.4–9.4	< 0.01

The categorical variables were MetS component (reference: none), number of MetS components (reference: number 0), Age at PCOS diagnosis (reference: adolescent), Elevated AST (reference: normal AST level at PCOS diagnosis), Elevated ALT (reference: normal ALT level at PCOS diagnosis), 2-h 75 g GTT insulin (reference: < 100 µU/mL), FAI (reference < 4.5), HA (reference: none).

*ALT* alanine aminotransferase, *AMH* anti-Müllerian hormone, *AST* aspartate transaminase, *CI* confidence interval, *FAI* free androgen index, *FGIR* fasting glucose insulin ratio, *HA* hyperandrogenism, *HDL* high-density lipoprotein, *HOMA-IR* homeostasis model assessment of insulin resistance, *HR* hazard ratio, *HTN* hypertension, *GTT* glucose tolerance test, *LH* luteinizing hormone, *MetS* metabolic syndrome, *NAFLD* non-alcoholic fatty liver disease, *PCOS* polycystic ovary syndrome, *SHBG* sex hormone binding globulin.

**Figure 4:**
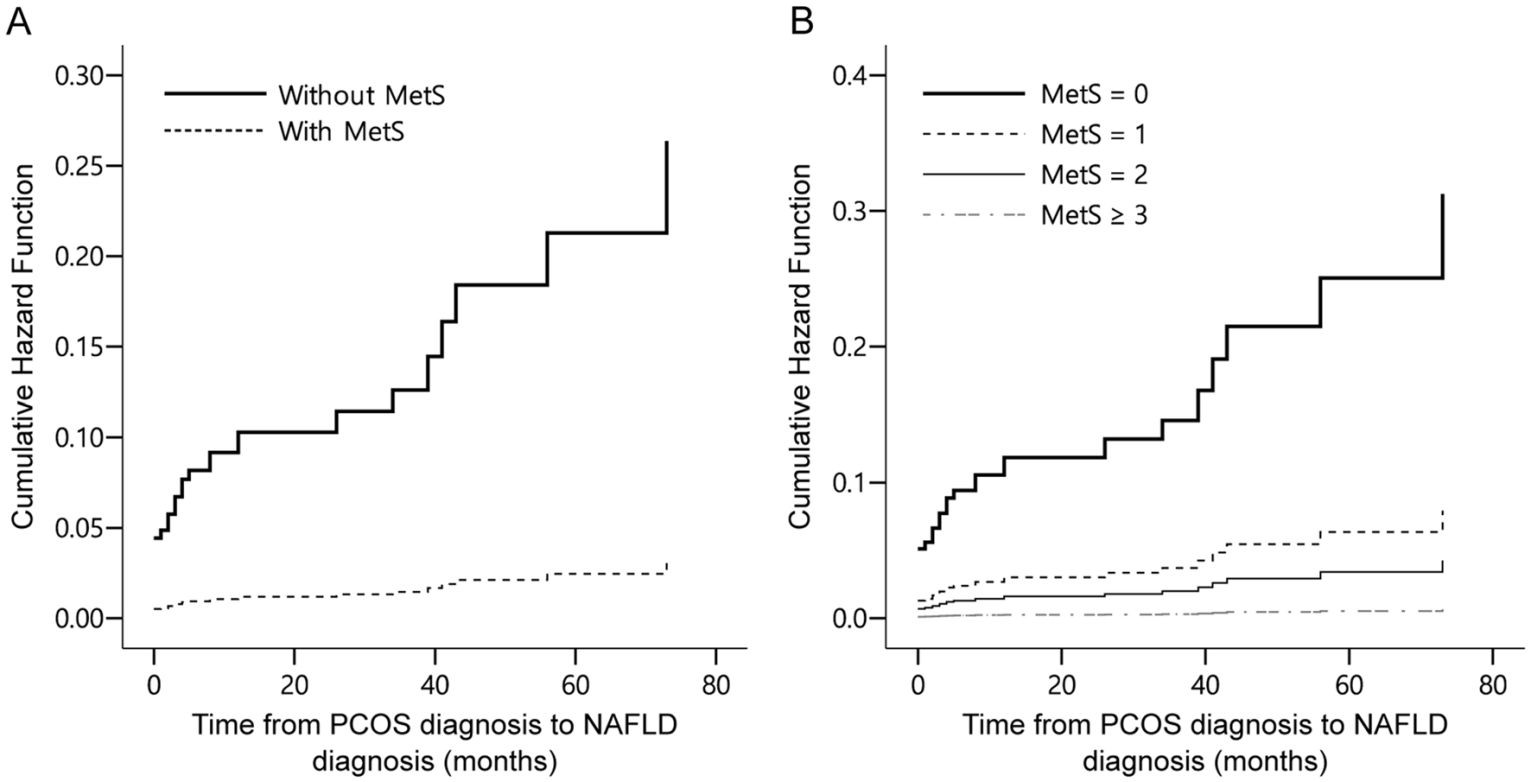
Cox regression analysis of non-alcoholic fatty liver disease diagnosis since polycystic ovary syndrome diagnosis (**a**) according to metabolic syndrome (MetS) diagnosis and (**b**) according to the number of MetS components in Korean women.

Multivariate cox analyses of variables associated with NAFLD were presented in two models (Table 4). The first model was according to MetS diagnosis, and the second model was according to the number of MetS components, which was not significant for a single component, but was significant for ≥ 3 components. AST level was significant in both models, while ALT level was not. Insulin resistance, represented as 2-h 75 g GTT insulin ≥ 100 μU/mL, was not significant in both models. Significance of HA also concurred in both models. BMI, both lean and obese groups were not significant compared to a reference BMI of 20–25 in both models. COX regression analysis of time from NAFLD diagnosis following PCOS diagnosis in months is shown in Fig. 4. The cumulative hazard function of NAFLD diagnosis following PCOS diagnosis was significantly higher when patients were diagnosed with MetS (Fig. 4a). It was also sequentially higher as the number of MetS components increased (Fig. 4b).

**Table 4:** Multivariate analysis of variables associated with NAFLD.

Variables	HR	95% CI	*p* value
**Model 1**			
MetS diagnosis	5.6	2.2–14.4	< 0.01
Elevated AST	8.5	2.8–25.6	< 0.01
Elevated ALT	1.4	0.5–4.3	0.52
2-h 75 g GTT insulin ≥ 100 µU/mL	1.2	0.5–2.5	0.70
HA	4.4	1.4–13.4	0.01
BMI 20–25 kg/m^2^ (Reference)			
BMI < 20 kg/m^2^ (Lean)	0.5	0.1–4.6	0.55
BMI > 25 kg/m^2^ (Obese)	2.2	0.6–8.0	0.24
**Model 2**			
Number of MetS components			
1	6.4	0.7–61.3	0.11
2	12.0	1.0–141.5	0.05
≥ 3	50.8	4.5–576.7	< 0.01
Elevated AST	7.8	2.7–22.5	< 0.01
Elevated ALT	1.6	0.5–4.6	0.42
2-h 75 g GTT insulin ≥ 100 µU/mL	1.1	0.5–2.3	0.89
HA	4.2	1.4–13.0	0.01
BMI 20–25 kg/m^2^ (Reference)			
BMI < 20 kg/m^2^ (Lean)	0.7	0.1–6.5	0.75
BMI > 25 kg/m^2^ (Obese)	0.9	0.2–3.5	0.82

(a) Model 1: according to MetS diagnosis. (b) Model 2: according to the number of MetS components.

The categorical variables were MetS component (reference: none), number of MetS components (reference: number 0), Elevated AST (reference: normal AST level at PCOS diagnosis), Elevated ALT (reference: normal ALT level at PCOS diagnosis), 2-h 75 g GTT insulin (reference: < 100 µU/mL), HA (reference: none), BMI (reference: 20–25).

*ALT* alanine aminotransferase, *AST* aspartate transaminase, *BMI* body mass index, *CI* confidence interval, *GTT* glucose tolerance test, *HA* hyperandrogenism, *MetS* metabolic syndrome, *PCOS* polycystic ovary syndrome, *SHBG* sex hormone binding globulin, *NAFLD* non-alcoholic fatty liver disease.

### Relationship between MetS diagnosis and liver steatosis according to age

Additional multivariate cox analyses of variables associated with NAFLD was done according to age at PCOS diagnosis (Table 5). The cox models showed that MetS diagnosis was a significant factor in consequent NAFLD development for adults, but not in adolescents. For elevated AST and ALT, there was contrasting results for the adolescent and adult group. In adolescents, both elevated AST and ALT was significant. In adults, only elevated AST was significant. 2-h 75 g GTT insulin ≥ 100 μU/mL was not a significant variable associated with NAFLD in both adolescents and adults. HA was significant in the adult group. It was not checked in the adolescent group as 100% of patients had HA. BMI was not significant for both adolescents and adults.

**Table 5:** Multivariate analysis of variables associated with NAFLD in adolescents and adults.

Variables	HR	95% CI	*p* value
**Model 1**			
MetS diagnosis	4.9	0.8–30.3	0.09
Elevated AST	5.5	1.1–28.4	0.04
Elevated ALT	23.8	1.9–292.9	0.01
2-h 75 g GTT insulin ≥ 100 µU/mL	1.5	0.2–9.3	0.69
BMI < 25 kg/m^2^ (Reference)			
BMI > 25 kg/m^2^ (Obese)	5.0	0.4–62.4	0.22
**Model 2**			
MetS diagnosis	5.2	1.6–16.4	< 0.01
Elevated AST	23.8	6.3–90.5	< 0.01
Elevated ALT	0.6	0.2–2.1	0.43
2-h 75 g GTT insulin ≥ 100 µU/mL	1.4	0.6–3.2	0.49
HA	6.1	1.8–20.1	< 0.01
BMI 20–25 kg/m^2^ (Reference)			
BMI < 20 kg/m^2^ (Lean)	0.4	0.1–4.1	0.45
BMI > 25 kg/m^2^ (Obese)	1.7	0.4–8.0	0.48

(a) Model 1: Adolescent group. (b) Model 2: Adult group.

The categorical variables were MetS component (reference: none), number of MetS components (reference: number 0), Elevated AST (reference: normal AST level at PCOS diagnosis), Elevated ALT (reference: normal ALT level at PCOS diagnosis), 2-h 75 g GTT insulin (reference: < 100 µU/mL, HA (reference: none), BMI (reference: < 25 kg/m^2^ in model 1, reference 20–25 kg/m^2^ in model 2).

*ALT* alanine aminotransferase, *AST* aspartate transaminase, *CI* confidence interval, *GTT* glucose tolerance test, *HR* hazard ratio, *MetS* metabolic syndrome, *NAFLD* non-alcoholic fatty liver disease, *PCOS* polycystic ovary syndrome, *HA* hyperandrogenism.

## Discussion

Our study suggests that metabolic disturbances are intimately related to the pathophysiology and development of liver disease in women with PCOS. While the separate components of MetS were not critically connected to NAFLD, the greater the number of MetS components, the higher the prevalence of NAFLD diagnosis and more severe was the liver fibrosis and steatosis. In previous studies determining the relationship between NAFLD and MetS, features of metabolic disturbances were frequently detected in NAFLD, leading to the theory that NAFLD is the hepatic expression of MetS^6^. Even though MetS diagnosis and the number of MetS components showed significant risk increase of developing NAFLD, obesity and insulin resistance itself did not turn out to be a direct risk factor in our results. Our cohort was composed of ethnically homogenous northeast Asian population with lean body mass^33^. Although obese cases were dominant in the NAFLD group compared to the non-NAFLD group, still the BMI was relatively lower than other studies’ population^25^. Generally, PCOS and NAFLD are representative metabolic diseases associated with insulin resistance^6^. The cut-offs of indices for insulin resistance and insulin sensitivity are controversial, especially in the non-diabetic population^5^. In the current study, we included 2-h 75 g GTT insulin, FGIR, and HOMA-IR. 2-h 75 g GTT insulin was the only index that may be applied commonly in both adolescent and adult. Although traditional references suggest a cut-off of 100 μU/mL as grading severe insulin resistance, a few studies questioned whether ethnicity, race may affect insulin sensitivity and end organ response^34^. Therefore, our study presents similar results to suggest that MetS is associated with NAFLD in PCOS women, rather than obesity itself, especially in the lean BMI population.

As HA was a prerequisite in diagnosing PCOS in adolescents as opposed to being one of three requirements in young adults, it could be the decisive factor associated with NAFLD in lieu of age. Previously, liver fat accumulation was associated with increasing age^35^. This was not found to be true in our investigation. Because NAFLD can arise regardless of age, instead of waiting for outward manifestations of liver injury to present, identification of risk factors at the time of PCOS diagnosis can lead to preventative actions. We found that HA, independent of insulin resistance and obesity, was significantly associated with NAFLD occurrence in accordance with previous studies^4,36^. This was consistent with a meta-analysis which showed that HA, was an independent factor associated with NAFLD in PCOS patients^18^.

In previous studies on women with PCOS, the prevalence of elevated liver enzyme levels was higher in women with PCOS than in controls^13^. The proportion of subjects with elevated ALT levels, which is more hepatocyte specific, was higher than that with elevated AST levels^13,15^. Interestingly, although both AST and ALT level elevations were associated with NAFLD in our univariate analysis, only AST was significant in our multivariate analysis. Up to 80% of all NAFLD patients have normal range ALT levels and it also decreases as liver fibrosis progresses to liver cirrhosis^37^, so the results of our study may actually be on par with the progression of NAFLD. Although elevated AST was significant in adolescents and elevated ALT was significant in adults, the actual serum AST/ALT levels were not very divergent. The number of adolescent PCOS patients was very small, so this conflicting result may not have any clinical significance. Liver enzyme levels are not always elevated and can be normal in NAFLD patients^38–41^. Liver fat accumulation evaluation through TE or abdominal ultrasonography was not done if there were no elevations in serum AST/ALT levels. Because this is not a defining feature of liver injury in patients with PCOS, patients presenting with MetS and HA should consider liver evaluation. Most current PCOS guidelines do not recommend for the screening of NAFLD^21,42^, but the duration of time that lapsed between the time of PCOS diagnosis to NAFLD diagnosis is approximately 3 years for both adolescents and adults. If adolescent PCOS patients are not routinely monitored, by the time the patient revisits a gynecologist to check up on their irregular menstruation, liver damage or other metabolic disturbances may have already progressed.

This study investigated NAFLD incidence in PCOS women and comprehensively examined all MetS components in relation to NAFLD. As liver biopsy was not performed in our study, the true prevalence of NAFLD and steatosis and fibrosis severity remain unknown. The limitations of this study stem from its retrospective design. Because not all patients had values available for all data items, the extent of potential relationships with NAFLD may have been diluted. A significantly higher number of adults were included in the study compared to adolescents, which could underestimate risk factors in the adolescent group. Except for incidentally found NAFLD, most of the patients were referred to the hepatology department for further examination due to liver enzyme abnormalities, and accordingly, there may have been selective bias in the diagnostic flow of NAFLD. Mild expressions of NAFLD without liver enzyme abnormality were not included, which contributes to the low prevalence of NAFLD in this study group. Waist circumference was not checked in most patients, so central obesity was not properly established. BMI was used as a substitute because studies published in Northeast Asian countries^25,43,44^ and even a large cross-sectional study of U.S. and Spanish populations^45^ showed BMI was correlated with metabolic impairment in lean PCOS patients. Further prospective studies of patients with MetS irregularities are warranted to determine whether patients without liver enzyme level elevations should undergo liver examinations.

In summary, our study results demonstrated that the diagnosis of MetS and HA were significant variables associated with NAFLD in women with PCOS. In light of the fact that MetS factors have a high correlation to NAFLD, patients presenting with metabolic disturbances at the time of PCOS diagnosis should be counseled to consider liver evaluation even if there are no outward manifestations of liver disease. Adding a cursory transabdominal ultrasound examination of the liver could be considered while doing a gynecologic ultrasound. PCOS manifests in women at reproductive age and evolves into metabolic problems with time. In the long run, NAFLD can have catastrophic liver-related mortalities if left untreated^46^. Earlier diagnosis can lead to more timely treatment. Although previous studies have shown the association between HA and MetS with NAFLD, we demonstrated in our study that adolescent PCOS patients, all of whom had HA, were susceptible to NAFLD when compounded with the diagnosis of MetS. Regardless of the age at which PCOS diagnosis was made, comorbidities can lead to an increased metabolic risk; thus, systematized investigation of NAFLD may be helpful.
